# Co‐occurrence of celiac disease and glycogen storage disease in a five‐year‐old patient with diabetes mellitus; a case report

**DOI:** 10.1002/ccr3.7804

**Published:** 2023-08-21

**Authors:** Sina Khani, Amirali Soheili, Seyed Mohammad Vahabi, Naghi Dara, Aliakbar Sayyari, Yalda Nilipour, Maryam Parvizi, Amirhossein Hosseini

**Affiliations:** ^1^ Medical Student Research Committee, School of Medicine Shahid Beheshti University of Medical Sciences Tehran Iran; ^2^ School of Medicine Tehran University of Medical Sciences Tehran Iran; ^3^ Pediatric Gastroentrology, Hepatology and Nutrition Research Center Research Institute for Children's Health Shahid Beheshti University of Medical sciences Tehran Iran; ^4^ Pediatric Pathology Research Center, Research Institute for children's Health Shahid Beheshti University of Medical Sciences Tehran Iran

**Keywords:** celiac disease, diabetes mellitus, glycogen storage disease, GSD III

## Abstract

A patient presented with edema, ascites and jaundice. Histologic report was consistent with Celiac Disease. Liver biopsy commensurate with Glycogen storage disease III, which was confirmed by genetic testing. A gluten‐free diet was initiated. After 2 months, ascites was relieved, hepatic function was improved, and hepatic size reduced.

## Graphical abstract

xxxx.

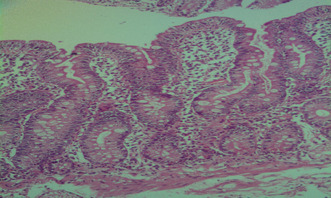

## 1 INTRODUCTION

Acute liver failure (ALF) is a scarce but mortal condition which develops in less than 8 weeks. Typical signs and symptoms of ALF are jaundice, bleeding disorder, and even hepatic encephalopathy in severe elderly cases. Although acute viral hepatitis is the most prevalent infantile cause of ALF, various etiologies are known to result in such condition including autoimmune hepatitis, infiltrative diseases, irradiation, and particularly metabolic disorders such as Glycogen storage disease (GSD).^1^, ^2^

Forbes‐Cori disease (GSD IIIb) is a rare autosomal recessive metabolic disorders occurred due to a defect in the AGL gene resulting in the abnormal activity of glycogen debranching enzyme (GDE) which is responsible to convert glycogens branches to glucose.^3^ This type of GSD is accounted for nearly 24% of GSDs, with an incidence rate of 1 per 80–100,000 in western countries. Naturally, muscles remain intact but, liver is often involved during the clinical course; though Liver cirrhosis is barely reported.^3^ Furthermore, patients most typically present in childhood with hepatomegaly, impaired fasting glucose, ketotic hypoglycemia, hyperlipidemia, and also failure to thrive (FTT).^3^, ^4^

Prediabetic state and diabetes mellitus are two common comorbidities or even results of this metabolic disorder, which are somehow complicated to treat if GSD remains undiagnosed.^3^ Likewise, other disease might accompany GSD in these patients.

Herein, we report an unusual coexistence of celiac disease with GSD in a diabetic 5‐year‐old boy which was a not only a rare comorbidity but also a challenging case to diagnose and manage. Failure to diagnose GSD can worsen the course of the disease. In these cases, the patient is hospitalized only with the diagnosis of diabetes and celiac disease and does not recover.

## 2 CASE PRESENTATION

**FIGURE 1:**
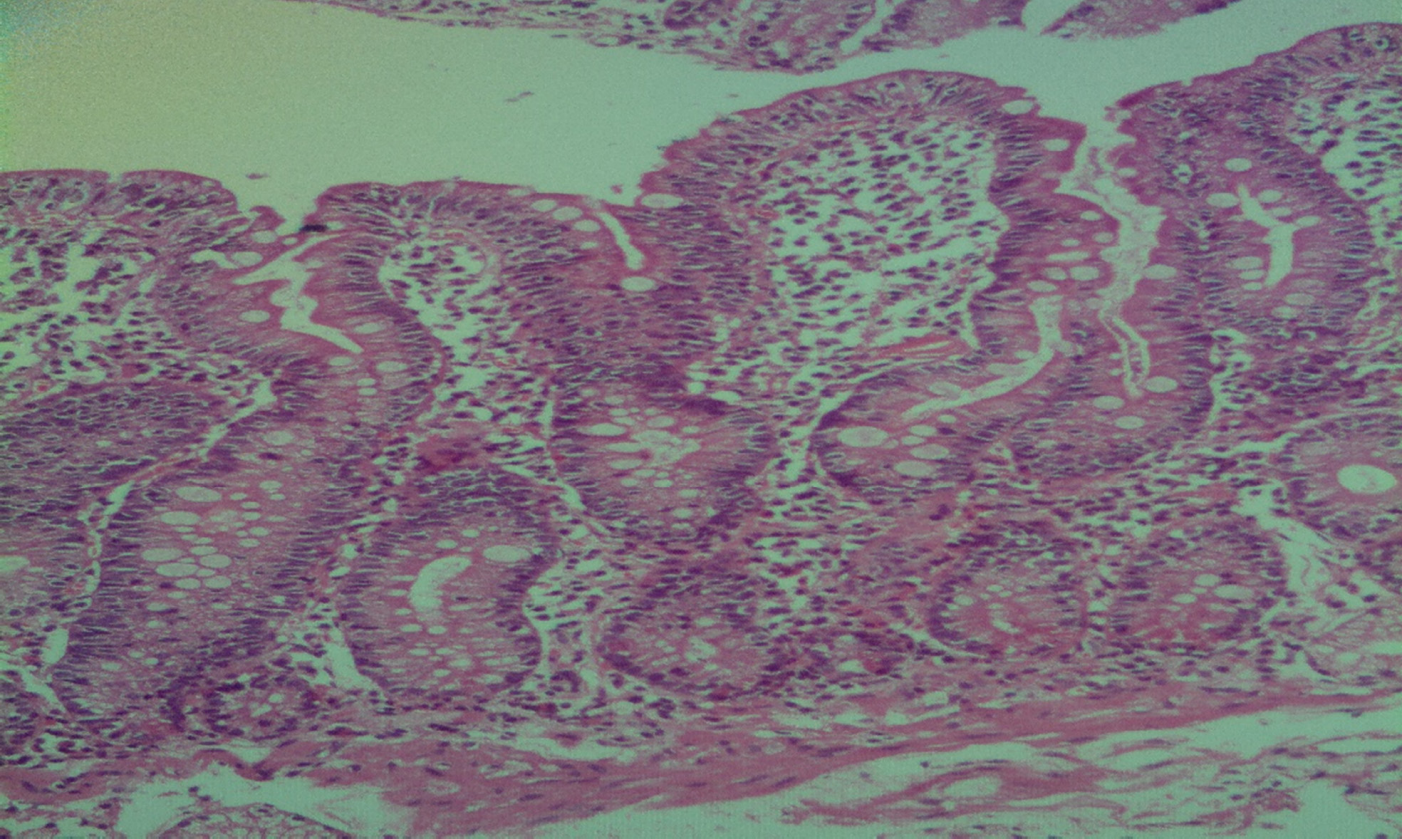
Endoscopic biopsy from duodenum revealed villi shortening with increased intraepithelial lymphocytes more than 40/100 enterocytes (Hematoxylin and Eosin x 400).

**FIGURE 2:**
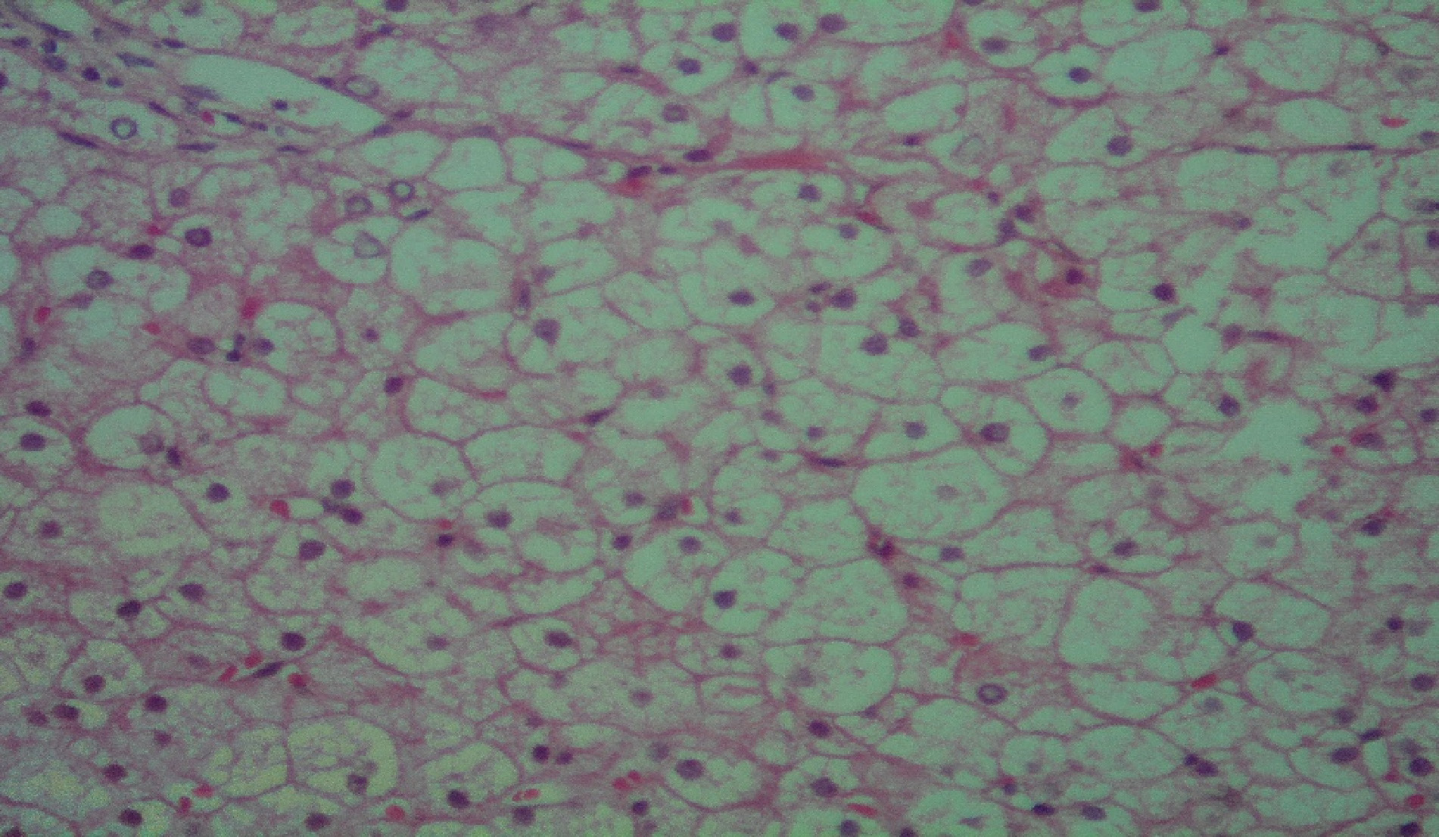
Liver needle biopsy showed mosaic pattern due to enlarged hepatocytes by glycogen accumulation compressing sinusoids. Glycogenated nuclei were also noted.

A 5‐year‐old boy with progressive generalized edema from 1 month ago, severe ascites, and jaundice was referred to Mofid children's hospital. The patient had diabetes mellitus as a preexisting medical condition and was receiving daily insulin injections. On presentation and physical examination, the patient had severe pitting edema in both upper and lower extremities, periorbital edema, icteric sclera, non‐tender firm hepatomegaly 4 cm below right costal margin and massive splenomegaly. Other parts of physical examination were relatively normal. Initial laboratory data is shown in Table 1. Initial abdominopelvic ultrasonography detected hepatomegaly (128 mm), splenomegaly (80 mm), and slight free fluid in abdomen. After initial resuscitation for hepatic failure and coagulopathy, upper esophagogastroduodenoscopy (EGD) was performed which showed normal esophagus, mild antral gastritis, and diffuse atrophic villi with scalloping in duodenum. Further histologic report from duodenal mucosal confirmed increased intraepithelial lymphocytes with mild villous atrophy and crypt hyperplasia (Figure 1), which was consistent with Celiac disease (Marsh 3a classification). Also, the patient was HLA‐DQ2 positive and had high serum Anti‐tTG and endomysial IgA. Due to high serum triglyceride‐cholesterol values and hepatosplenomegaly and associated hepatic failure coagulopathy; liver needle biopsy was done. Histopathologic results included hepatocytes with strong positivity for glycogen (Periodic Acid Schiff positive) without inflammation, cholestasis or fibrosis (Figure 2), which was in the clinical context of patient compatible with GSD type III. By doing genetic test, the diagnosis of GSD III was confirmed for the patient. According to these clinical diagnoses, the patient was put on a gluten‐free diet with uncooked cornstarch. Also, the patient received zinc and multivitamins daily. He received insulin as before. After 2 months of follow‐up evaluation coagulopathy was ameliorated, ascites was relieved, hepatic function was improved and hepatic size reduced. The patient also showed reduced need for insulin and better glycemic control was achieved.

**TABLE 1:** Initial laboratory data of the patient.

Day of admission lab values	1st	6th	12th
WBC	5.3	11.1	12.1
RBC	3.7	3.55	3.14
Hb	10.9	10.4	8.9
HCT	34.5	32.6	29.4
PLT	583	674	868
Na	133	138	137
K	4.5	5.3	4.3
Ca	7.2	8.3	8.4
Mg	2.0	1.6	1.7
Bilirubin T	1.1	0.5	0.4
Bilirubin D	0.1	0.15	0.10
ALP	328	402	611
AST	155	1238	300
ALT	268	826	544
Triglyceride	281	417	
BS	215	269	165
BUN	17.9	8.1	25
Creatinine	0.57	0.5	0.6
Total Protein	5.4	6.6	7.3
Serum Alb	3.3	3.9	4.7
PT	12	12	12
PTT	25	25	28
INR	1	1	1
IgA (Eliza)	262	260	267
IgG (Eliza)	629		

## 3 DISCUSSION

Celiac disease is a systemic autoimmune medical condition characterized by various serologic and histopathologic features; triggered by gluten‐containing foods in genetically susceptible patients.^5^ It is more commonly pronounced in women with a female to male ratio of 1.5 to 2:1 and has an overall prevalence of 0.4% to 1.3% in the pediatric population.^6^ The disease presents with various gastrointestinal (including diarrhea, vomiting, weight loss, and abdominal distension) and systemic manifestations; of which the former being more common in children and the latter being more common in the adult population.^7^ Tissue transglutaminase (tTG) IgA antibody and endomysial IgA antibody (EMA) have the highest sensitivity and specificity in both children and adults in diagnosing the disease but the endoscopic biopsy of the small intestine with histologic criteria remains the gold standard.^6^ On the other hand, Type III GSD is an autosomal recessive disease with loss of function mutations of the GDE and characterized by the storage of abnormal glycogen in both skeletal and cardiac muscle and/or the liver. It has various prevalence among many different ethnic groups and with clinical features of hypoglycemia, pronounced hepatomegaly and cirrhosis, hyperlipidemia, variable skeletal myopathy, variable cardiomyopathy, and poor growth. Also, the disease has different subtypes based on the enzymatic and clinical picture, which is beyond our scope of work in this report. Although the histologic findings in patients with GSD III may be diagnostic, the only biochemical abnormality is the elevation of the serum AST and ALT concentrations which is accompanied by normal liver synthetic function including serum albumin, PT, and bilirubin concentrations.^4^ We did not find any reports of a patient having the three diseases of celiac, GSD type III and DM in the literature but a case report from Egypt in 2009 described a 19‐year‐old female patient which was a known case of GSD type III from two and half years‐old and recently had developed DM. They concluded that insulin therapy was superior to oral hypoglycemic agents in controlling the DM and recommended serial oral glucose tolerance tests (OGTT) for early detection and management of glucose intolerance in GSD type III because of disease nature which patients are prone to hypoglycemia.^8^ Another report included a 10‐month‐old boy which was diagnosed with GSD type III and treated with meals low in carbohydrates and high in protein (to stimulate gluconeogenesis) and uncooked corn starch. Unfortunately, his clinical symptoms even worsened, and he developed more abdominal distention and steatorrhoeic stools, which prompt a search for the cause of malabsorption. In further studies, he was diagnosed with celiac disease and put on a gluten‐free diet in addition to his past diet, which later on follow‐ups improved rapidly and gained weight normally.^9^

## 4 CONCLUSION

Although co‐occurrence of celiac disease and GSD is rare; diagnosis of celiac disease can play an important role in the treatment of these patients. A proper diet can improve the symptoms of patients and even reduce the patients' need for insulin.

## AUTHOR CONTRIBUTIONS

**Sina Khani:** Writing – original draft; writing – review and editing. **Amirali Soheili:** Writing – original draft. **Seyed Mohammad Vahabi:** Writing – review and editing. **Naghi Dara:** Data curation; supervision. **Aliakbar Sayyari:** Investigation. **Yalda Nilipour:** Data curation; writing – review and editing. **Maryam Parvizi:** Data curation; writing – review and editing. **Amirhossein Hosseini:** Conceptualization; data curation; supervision.

## FUNDING INFORMATION

The authors did not receive any fundings for this study.

## CONFLICT OF INTEREST STATEMENT

None of the authors of this article have a conflict of interest in this field.

## CONSENT

Written informed consent was obtained from the patient's father to publish this report in accordance with the journal's patient consent policy.

## Data Availability

The data that support the findings of this study are available from the corresponding author upon reasonable request.
